# Endotoxin tolerance inhibits NLRP3 inflammasome activation in macrophages of septic mice by restoring autophagic flux through TRIM26

**DOI:** 10.1515/med-2025-1231

**Published:** 2025-09-03

**Authors:** Yanyan Yang, Shiwen Wu, Minghao Lin, Xueting Xie, Huifang Shi, Youran Chen, Shanshan Li, Yuchun Jiang, Sijie Zheng, Chibin Shen, Naibin Yang, Mingqin Lu

**Affiliations:** Department of Infectious Diseases, The First Affiliated Hospital of Wenzhou Medical University, Wenzhou, Zhejiang, China; Department of Ultrasonography, Nanjing Hospital of Traditional Chinese Medicine, Nanjing, Jiangsu, China; Department of Laboratory Medicine, The First Affiliated Hospital of Ningbo University, Ningbo, Zhejiang, China; Department of Infectious Diseases, The First Affiliated Hospital of Ningbo University, No. 59 Liuting Road, Ningbo, Zhejiang, China; Consortium for Infection and Innovation (CII), The First Affiliated Hospital of Wenzhou Medical University, Wenzhou, Zhejiang, China

**Keywords:** sepsis, endotoxin tolerance, autophagic flux, NLRP3, TRIM26

## Abstract

**Objective:**

Endotoxin tolerance (ET) has been demonstrated to attenuate the inflammatory response in murine models of sepsis. This study seeks to elucidate the underlying mechanisms by which ET modulates inflammation in sepsis, with a particular focus on macrophage autophagy.

**Methods:**

An *in vivo* sepsis model was generated using cecal ligation and perforation, while an *in vitro* model of inflammatory injury was induced via lipopolysaccharide (LPS) administration. ET was established through pretreatment with low-dose LPS. Subsequent analyses were conducted to assess the presence of the NLRP3 inflammasome, autophagic flux, and the expression levels of TRIM26.

**Results:**

Heightened inflammation was observed in the TNF-α levels and various organs of the sepsis group; conversely, inflammation was reduced in the group receiving ET treatment. Upon stimulation with LPS, primary mouse peritoneal macrophages exhibited activation of the NLRP3 inflammasome and autophagy, accumulation of mitochondrial reactive oxygen species, compromised membrane potential, resulting in cell apoptosis, and decreased expression of TRIM26. ET was found to enhance autophagy, suppress the activation of NLRP3 inflammasomes, and upregulate the expression of TRIM26. Interestingly, modulation of autophagy levels either reversed or intensified the protective effects of ET on macrophages *in vitro*. Knockdown of TRIM26 using small interfering RNA (siRNA) resulted in increased NLRP3 inflammasome activation and accumulation of P62.

**Conclusion:**

We reveal that ET restores the autophagic flux in macrophages, inhibit NLRP3 inflammasome activation, and mitigate inflammatory damage in septic mice, potentially through the regulation of TRIM26.

## 1 Introduction

Sepsis, a condition characterized by life-threatening organ dysfunction resulting from a host’s exaggerated response to infection, presents challenges in both diagnosis and treatment due to its intricate pathogenesis [1,2]. Therefore, it is essential to thoroughly comprehend the pathogenesis of sepsis and explore therapeutic approaches. Macrophages are pivotal in the progression of sepsis, with research indicating the significant involvement of peritoneal macrophages in altering immune function in septic mice [3]. The activation of the NLRP3 inflammasome is implicated in the pathogenesis of various inflammatory conditions, for example sepsis, leading to significant elevations in IL-1β, IL-18, and caspase-1 levels [4]. Previous research has demonstrated that a substantial quantity of NLRP3 accumulates within macrophages, which in turn directly mitigate the production of reactive oxygen species (ROS) and the release of mitochondrial DNA through mitophagy, consequently impeding NLRP3 activation [5].

Autophagy has been identified to safeguard immune status by modulating macrophage functions, such as phagocytosis, antigen presentation, secretion of inflammatory factors, and clearance of apoptotic lymphocytes [6,7]. In sepsis, autophagy plays a protective role by modulating macrophage activation and polarization to mitigate inflammasome activation and the release of inflammatory mediators [8,9]. Further investigations have revealed that during sepsis, macrophage autophagy hinders the activation of the NLRP3 inflammasome by targeting the lysosome responsible for degrading IL-1β, thereby diminishing the secretion of IL-1β [10]. Conversely, deficiencies in macrophage autophagy may lead to heightened abnormal NLRP3 activation and the initiation of inflammatory reaction [11]. Therefore, targeting macrophage autophagy represents a novel therapeutic approach for the management of sepsis.

The most precise method for evaluating autophagy levels involves assessing autophagic flux, which encompasses the entire dynamic process from the formation of autophagosomes to their fusion with lysosomes [12]. TRIM26, a crucial E3 ubiquitin ligase within the tripartite motif (TRIM) family, plays a significant role in various pathophysiological processes including innate immune regulation, microbial suppression, and tumorigenesis [13]. Belonging to the RING family of E3 ubiquitin ligases, this tripartite motif protein consists of RING, B-Box, coil-coiled, and C-terminal domains [14]. Members of the TRIM family have the ability to interact with inflammasomes and facilitate their binding to p62, ultimately leading to inflammasome degradation through autophagy. Wang et al. discovered that TRIM26 exerts a negative regulatory effect on interferon-β production and antiviral immune responses by means of polyubiquitination and subsequent degradation of nuclear IRF3 [13]. Given the pivotal role of TRIM26 in the antiviral infection process, it is plausible that this protein may also be implicated in the inflammatory response, particularly in the activation of NLRP3 inflammasomes.

Endotoxin tolerance (ET) is characterized by a state of hyporesponsiveness or unresponsiveness to re-stimulation with high-dose endotoxins following prior continuous exposure to low-dose endotoxins [15]. ET is associated with upregulation of CD64 receptors on macrophage surfaces, downregulation of MHC class II molecules, modulation of inflammatory cytokines, upregulation of anti-inflammatory cytokines, enhancement of phagocytic activity and pathogen removal, and reduction of the effect of antigen presentation, thereby reducing specific immune damage [15]. The proposal of utilizing ET as a preventive treatment to mitigate mortality resulting from endotoxemia is grounded in its role as a protective mechanism against lipopolysaccharide (LPS)-induced inflammation. Nevertheless, the precise mechanisms through which ET orchestrates alterations in immune function remain to be fully elucidated.

Here, this study aims to investigate the potential mechanism of ET in reducing inflammation in sepsis based on macrophage autophagy and reveal whether ET restores the autophagic flux in macrophages, inhibit NLRP3 inflammasome activation in septic mice through the regulation of TRIM26.

## 2 Materials and methods

### 2.1 Animals and materials

A total of 48 SPF-grade male C57BL/6 mice, weighing 20–25 g and aging 6–8 weeks, were purchased from the Shanghai Experimental Animal Center of the Chinese Academy of Sciences and raised in the Experimental Animal Center of Wenzhou Medical University. The Animal Policy and Welfare Committee of Wenzhou Medical University approved all animal care and experimental procedures (Approval Document No. Wydw2020-0838). All animal experiments were performed at the Animal Experiment Center of Wenzhou Medical University.

### 2.2 Establishment of sepsis model *in viv*o

The experimental mice were randomly divided into three groups, including sham operation group (*N* = 6), sepsis group (*N* = 6), and ET+ sepsis group (*N* = 6). The sepsis model *in vivo* was established through cecal ligation and perforation (CLP). The ET+ sepsis group was intraperitoneally injected with a small dose of LPS (L4391, Sigma-Aldrich, USA) (0.1 mg/kg) every day for 5 days before CLP, while the sham operation group and sepsis group were intraperitoneally injected with 0.9% sodium chloride solution (7647-14-5, Sigma-Aldrich, USA) (0.1 mg/kg) every day for 5 days before the sham operation or CLP, respectively. The mice were killed after anesthetic treatment at 24 h after the operation, blood was collected from the orbit, and the liver, kidney, and ileum tissues were taken.

### 2.3 Histopathological analysis

The liver, kidney, and ileum tissues were fixed with 4% paraformaldehyde for 24 h, gradually dehydrated with ethanol, treated with xylene, and embedded in paraffin. Tissue wax block was cut into 4 μm-thick slices and placed on glass slides for hematoxylin and eosin (HE) staining (Cat#: G1120, Solarbio, China). HE staining was performed according to the manufacturer’s instructions. After mounting, slides were examined under a microscope (Olympus, Japan) to observe lymphocyte infiltration, fibrosis, and cell necrosis in each tissue.

### 2.4 Detection of inflammatory factors in serum

Serum was obtained from blood samples after centrifuging at 1,500 rpm for 10 min and immediately frozen for further analysis. ELISA kit (Multi Sciences, Hangzhou, China) was used to detect serum TNF-α (Cat No. EK282HS; Multi Sciences) and IL-10 (Cat No. EK210; Multi Sciences) concentrations, and to detect TNF-α (Cat No. EK282HS; Multi Sciences), IL-18 (Cat No. EK218EGA; Multi Sciences), and IL-1β (Cat No. EK201B; Multi Sciences) in collected cell culture supernatant, the manufacturer’s instructions were followed.

### 2.5 Isolation of primary peritoneal macrophages

Primary peritoneal macrophages were isolated as previously described [16,18]. Male C57BL/6J mice aged 6–8 weeks were intraperitoneally injected with 1 mL of 3% thioglycollate broth once daily for 3 consecutive days to induce sterile inflammation and promote the recruitment of macrophages to the peritoneal cavity. Three days after the final injection, mice were anesthetized with 2% isoflurane and euthanized by cervical dislocation.

The abdominal surface was sterilized by immersion in 75% ethanol for 3 min. Mice were then transferred to a biosafety cabinet, placed in the supine position, and fixed on a sterile surgical drape. The abdominal skin was gently lifted with forceps and incised in a “Y”-shaped pattern to expose the peritoneum, followed by a second disinfection with 75% ethanol.

Next, 5 mL of pre-chilled sterile saline was gently injected into the peritoneal cavity. The abdomen was softly massaged for 3–5 min to allow cells to dislodge into the lavage fluid. The peritoneal fluid was then carefully aspirated using a sterile syringe and the lavage procedure was repeated once to maximize cell recovery.

The combined peritoneal lavage fluid was centrifuged at 1,000 rpm for 5 min at room temperature. The supernatant was discarded, and the cell pellet was resuspended in RPMI-1640 medium. Cells were seeded in culture dishes and incubated at 37°C for 2 h. Non-adherent cells were removed by gentle washing, and the remaining adherent cells were considered purified primary peritoneal macrophages.

### 2.6 Cell culture

Primary peritoneal macrophages were extracted from healthy mice. Subsequently, low-dose LPS (100 ng/mL) stimulation was provided for 24 h to induce ET *in vitro* and high-dose LPS (1 μg/mL) stimulation was provided for 24 h. The mouse macrophage cell line RAW264.7 was obtained from the cell bank of the Chinese Academy of Sciences (Shanghai, China). The RAW264.7 cells were cultured in Dulbecco’s Modified Eagle Medium (DMEM; Gibco, USA) containing 10% fetal bovine serum (Gibco, USA) and subcultured in a 5% CO_2_ incubator at 37°C. At approximately 70% fusion, low-dose LPS (100 ng/mL) stimulation was provided for 24 h *in vitro* to induce ET, followed by pretreatment with 3-MA (Cat No. HY-19312; MedChem Express, USA) (5 μmol/mL) for 4 h or rapamycin (Cat No. HY-10219; MedChem Express, USA) (10 μmol/mL) for 24 h, and high-dose LPS (1 μg/mL) stimulation for 24 h.

### 2.7 Cell transfection

RAW264.7 cells were subcultured to 30–40% confluence, stimulated with a small dose of LPS (100 ng/mL) for 24 h to induce ET, and then transfected with control (si-NC, 5′-UUCUCCGAACGUGUCACGUdTdT-3′, 5′-ACGUGACACGUUCGGAGAAdTdT-3′) and TRIM26-small interfering RNA (siRNA) (5′-CCAGGAGAAGCUACACUACdTdT-3′, 5′-GUAGUGUAGCUUCUCCUGGdTdT-3′) using Gene Pharma siRNA-Mate Hanbio Technology (Shanghai, China). The cell culture medium was replaced with DMEM basal medium, and the cells were cultured for 6 h. After transfection, the cells were cultured in complete DMEM for 36 h. Stimulation with a large dose of LPS (1 μg/mL) for 24 h was used to induce sepsis.

### 2.8 Annexin V/PI double staining to detect macrophage apoptosis

Apoptosis of liver tissues in each group was assessed using Annexin V-fluorescein isothiocyanate (FITC)/PI Apoptosis Detection Kit (Cat No. AT101; Multi Sciences, China) according to the manufacturer’s instructions. Cells were collected from each group and washed twice with phosphate buffered saline (PBS) to obtain a macrophage suspension of 0.5 × 10^6^/tube. Subsequently, FITC-labeled Annexin V staining solution (10 μL) was added to the macrophage solution, mixed well, and incubated for 15 min at 22°C in the dark. Samples were centrifuged at approximately 1,000 rpm for 5 min at 4°C and the supernatant was discarded. Subsequently, the cells were resuspended in PBS and 5 μL of PI staining solution was added to the suspension. Samples were incubated at room temperature for 5–15 min in the dark, mixed well, and immediately detected on a flow cytometer (BECKMAN COULTER, USA), FlowJo software (Becton, Dickinson & Company, USA) was used to analyze the rate of apoptosis.

### 2.9 Measuring mitochondrial ROS (mtROS)

mtROS levels were measured using the Mito-Sox Red (Cat No. M36007; Thermo Fisher Scientific, USA). The macrophages were incubated with Mito-Sox Red (5 μm) for 30 min, washed three times with PBS, and observed under a fluorescence microscope (Olympus, Japan).

### 2.10 MitoTracker Red staining

MitoTracker Red CMXRos (Cat No. M7512; Thermo Fisher Scientific, USA) was used to detect live-cell mitochondria. The LPS-treated macrophages were incubated with 50 nM MitoTracker probe at 37°C for 30 min. Subsequently, the macrophages were washed three times with PBS and observed under a fluorescence microscope (Olympus, Japan).

### 2.11 Western blot analysis

Total cellular protein was extracted with lysis buffer (Solarbio, China) and quantified using a BCA protein analysis kit (Beyotime, China). After thermal denaturation at 100°C for 10 min, 50 μg of cell lysates were separated by 10% sodium lauryl sulfate-polyacrylamide gel electrophoresis and transferred to a polyvinylidene fluoride membrane (Millipore, Massachusetts, USA). Subsequently, the membrane was incubated with primary antibodies against NLRP3 (1:1,000, Cat No. 30109-1-AP; Proteintech), P62 (1:1,000, Cat No. 18420-1-AP; Proteintech), TRIM26 (1:1,000, Cat No. 13773T; CST), BECLIN1 (1:1,000, Cat No. A7353; ABclonal), LC3II (1:1,000, Cat No. ab192890; Abcam), ASC (1:1,000, Cat No. A22046; ABclonal), and GAPDH (1:1,000, Cat No. 60004-1-Ig; Proteintech) at 4°C overnight, and then conjugated with horseradish peroxidase (1:1,000, Cat No. A0208/A0216; Beyotime, China) for 1 h at room temperature. The bands were visualized using an enhanced chemiluminescence reagent (Bio-Rad Laboratories, USA).

### 2.12 Statistical analysis

Statistical analyses were performed using Prism software (version 9.0; GraphPad, La Jolla, CA, USA). Data are expressed as mean ± SEM. For multiple group comparisons, a one-way analysis of variance using Tukey’s or Dunnett’s *post-hoc* tests was performed. Values of *P* < 0.05 were considered to be statistically significant.


**Ethical approval:** The Animal Policy and Welfare Committee of Wenzhou Medical University approved all animal care and experimental procedures (Approval Document No. Wydw2020-0838).

## 3 Results

### 3.1 ET reduces the pathological inflammatory damage of organs in septic mice

**Figure 1:**
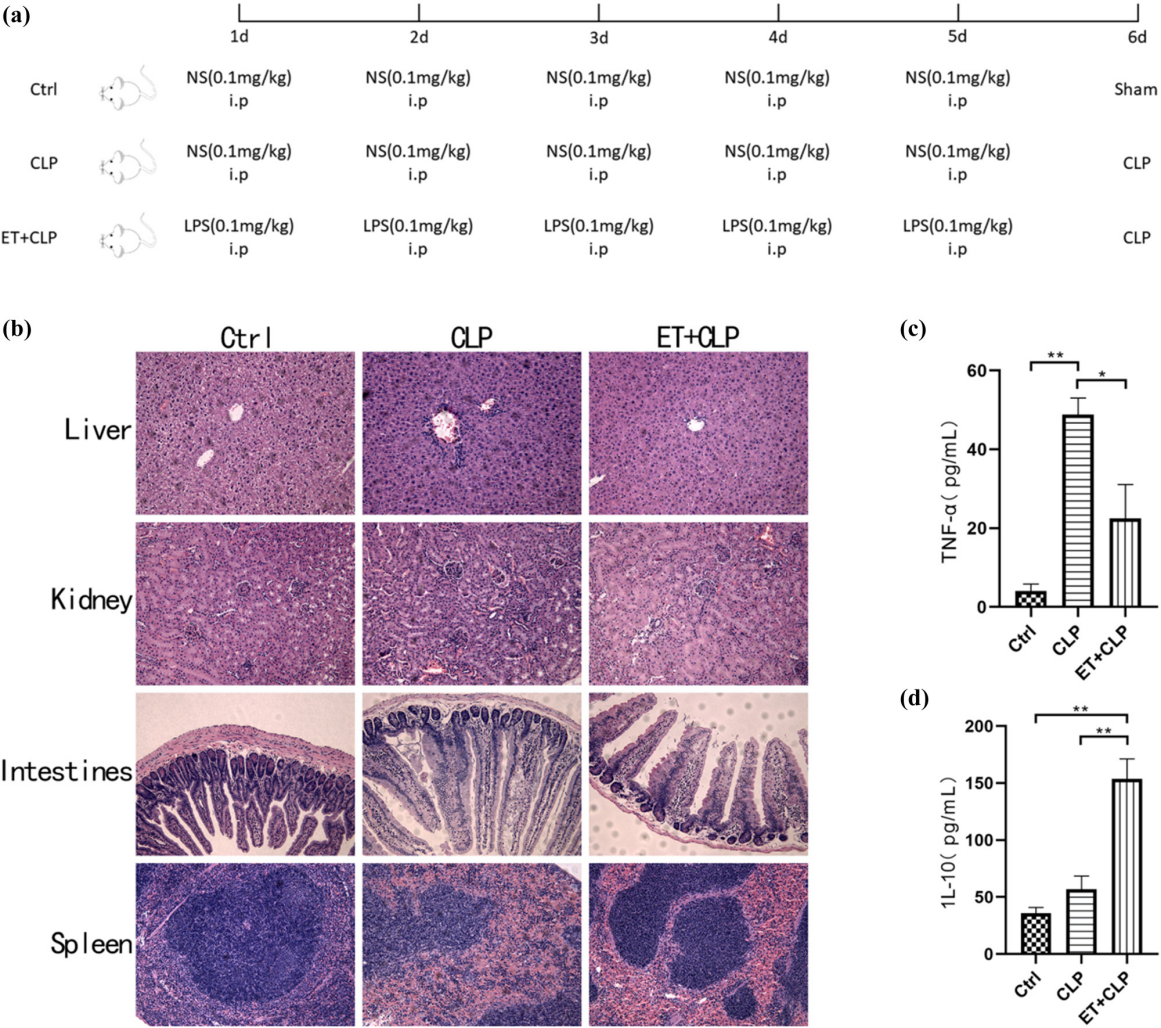
ET reduces the pathological inflammatory damage of organs in septic mice. (a) Establishment of sepsis mouse model and the time point of ET to intraperitoneal injection. (b) HE staining of liver, kidney, intestine, and spleen tissues (200×). (c) Serum TNF-α level by ELISA. (d) Serum IL-10 level by ELISA. *P < 0.05, **P < 0.01.

To investigate the effects of ET on septic mice, we first established a sepsis model and induced ET through intraperitoneal injection of a low dose of LPS (Figure 1a shows the model construction process). Compared to the sham-operated group, septic mice exhibited significant pathological changes: hepatocyte edema, degeneration, and disordered arrangement, along with liver lobule damage; increased glomerular volume, swollen and necrotic renal tubular epithelial cells, and dilated renal tubules with inflammatory cell infiltration; ileal edema and vacuolar degeneration, villi damage, and inflammatory cell infiltration; and disordered white pulp structure, blurred junction between red and white pulp, and red pulp congestion in the spleen (Figure 1b). Following ET treatment, these pathological changes were significantly alleviated: hepatocyte edema decreased, cell arrangement normalized, and liver lobule structure was partially restored; glomeruli slightly enlarged, with reduced renal tubular edema compared to the sepsis group; mild ileal edema with intact villi structure; and the spleen structure remained mostly intact with mild red pulp congestion. Inflammatory cell infiltration was reduced in all tissues, leading to a marked reduction in pathological damage (Figure 1b).

TNF-α, a key inflammatory mediator produced in response to stimuli such as viruses and LPS, is significantly elevated in septic patients [19]. IL-10, an anti-inflammatory cytokine, reduces excessive inflammation by supporting T cells and inhibiting the overexpression of pro-inflammatory cytokines such as IL-6 and TNF-α [20]. ELISA analysis revealed that serum TNF-α levels were significantly higher in the CLP group compared to the sham-operated group. Moreover, ET treatment not only reduced serum TNF-α levels but also significantly increased IL-10 concentrations in septic mice. These findings suggest that ET modulates immune responses, effectively mitigating the systemic inflammation and tissue damage induced by sepsis (Figure 1c and d).

### 3.2 ET inhibits NLRP3 inflammasomes activation in peritoneal macrophages in septic mice

The results from the *in vivo* experiments indicate that ET significantly lessens tissue pathological damage in septic mice and reduces serum inflammatory cytokine levels. To further investigate the underlying mechanisms, we established an *in vitro* model using primary mouse peritoneal macrophages. These cells were pretreated with a low dose of LPS (100 ng/mL) for 24 h, followed by three PBS washes and stimulation with a high dose of LPS (1 μg/mL) for an additional 24 h to simulate septic inflammation. Cells pretreated with LPS (100 ng/mL) were considered to have developed ET.

**Figure 2:**
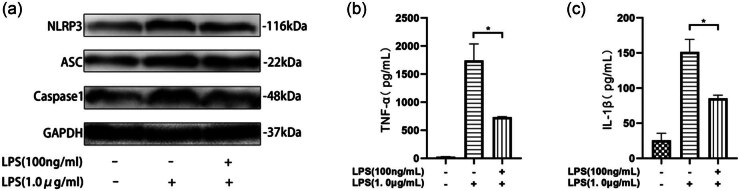
ET inhibits the activation of NLRP3 inflammasomes in peritoneal macrophages in septic mice. The primary mouse peritoneal macrophages were extracted and pretreated with LPS (100 ng/mL) for 24 h, washed with PBS three times, and then stimulated with LPS (1 µg/mL) for 24 h. The cells pretreated with LPS (100 ng/mL) were endotoxin-resistant cells, and LPS (1 µg/mL) stimulated the cells to mimic the inflammatory damage of peritoneal macrophages in septic mice. (a) Western blot analysis of the protein expression levels of NLRP3, ASC, and Caspase-1 in macrophages. (b) Levels of TNF-α and IL-1β in the cell culture supernatant by ELISA. (c) Levels of TNF-α and IL-1β in the cell culture supernatant by ELISA. *P < 0.05, **P < 0.01.

TNF-α downregulation across ET models has been widely reported, establishing it as a reliable biomarker for assessing ET [21,23]. In line with this, we used TNF-α levels as an indicator of ET in this study. Compared to controls, the LPS-pretreated cells exhibited a marked reduction in TNF-α secretion after re-stimulation with LPS (1 μg/mL) (Figure 2b), confirming the establishment of ET.

To explore the mechanisms involved, we focused on the recently identified PLK1/AMPK/DRP1 signaling axis, which has been shown to inhibit ROS-mediated NLRP3 inflammasome activation and attenuate sepsis-induced acute lung injury [24]. Aberrant NLRP3 activation has been implicated in sepsis-associated organ dysfunction, including encephalopathy [25,26], cardiomyopathy [27,28], and lung injury, with excessive activation exacerbating inflammation and leading to tissue damage and organ failure [29]. Thus, NLRP3 inflammasome activation is crucial to the pathogenesis of sepsis.

We evaluated the expression of NLRP3 inflammasome components, finding that LPS (1 μg/mL) treatment led to significantly elevated levels of NLRP3, ASC, and caspase-1 (Figure 2a), indicative of inflammasome activation. Additionally, IL-1β levels in the supernatants were markedly increased (Figure 2c), further supporting the role of NLRP3 activation in the inflammatory response.

Notably, ET (LPS 100 ng/mL pretreatment) resulted in significantly lower expression of NLRP3, ASC, and caspase-1, as well as reduced IL-1β secretion, compared to the LPS-only treatment group (Figure 2a and c). These findings suggest that ET may mitigate macrophage inflammatory damage by inhibiting NLRP3 inflammasome activation, thereby exerting protective effects. This provides novel experimental evidence for the anti-inflammatory mechanisms of ET in sepsis and opens potential therapeutic avenues for managing septic inflammation.

ET restores the autophagic flux of peritoneal macrophages in septic mice, reduces the production of mtROS, and inhibits macrophage apoptosis.

**Figure 3:**
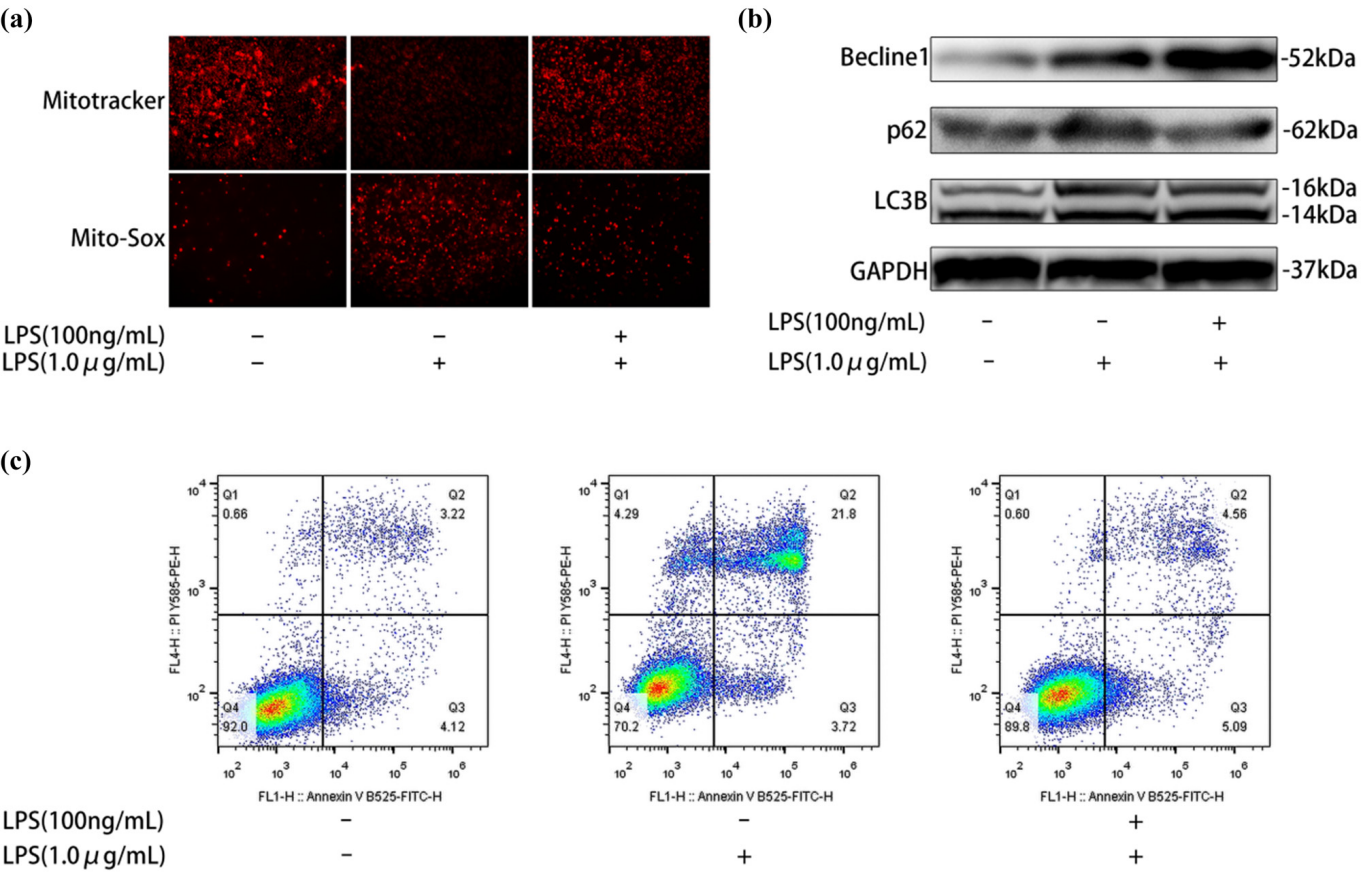
ET can restore the autophagic flux of peritoneal macrophages in septic mice, reduce the production of mtROS, and inhibit macrophage apoptosis. (a) MitoTracker Red probe was used to measure mitochondrial membrane potential and Mito-Sox was used to measure macrophage mtROS. (b) Western blot analysis of Beclin1, P62, and LC3B autophagy-related protein expression levels in macrophages. (c) Annexin V/PI double staining to detect macrophage apoptosis.

The activation of the NLRP3 inflammasome is regulated by multiple factors [30], among which autophagy [31], apoptosis [32], and mtROS have garnered considerable attention [33,34]. Therefore, we examined mtROS, apoptosis, and autophagy in macrophages stimulated with LPS (μg/mL) [35]. Our results showed that LPS stimulation markedly increased mtROS levels in macrophages and significantly impaired mitochondrial membrane potential (Figure 3a). Flow cytometric analysis further revealed a significant increase in apoptosis in LPS-treated macrophages compared with the control group (Figure 3c). In addition, western blot analysis demonstrated that LPS not only promoted apoptosis but also induced autophagy in macrophages (Figure 3b). However, in macrophages stimulated by LPS, LC3B-II/LC3B-I and Beclin-1 were increased, p62 accumulated, and autophagic flux was blocked. These results suggest that macrophages stimulated by LPS exhibit mitochondrial damage, increased mtROS production, increased apoptosis, and activated autophagy. We found that the responsiveness of macrophages pretreated with LPS (100 ng/mL) to LPS (1 μg/mL) stimulation was significantly lower than that of unpretreated cells. This is because of the generation of mtROS, damage to mitochondrial transmembrane potentials, and apoptosis of macrophages. However, LC3B-II/LC3B-I and Beclin-1 further increased, and the decrease in p62 indicated the restoration of autophagic flux. In summary, ET restored the autophagic flux of peritoneal macrophages in septic mice, reduced mtROS production, and inhibited macrophage apoptosis.

### 3.3 Inhibition of autophagy can reverse the protective effect of ET on LPS-stimulated RAW264.7 cells

**Figure 4:**
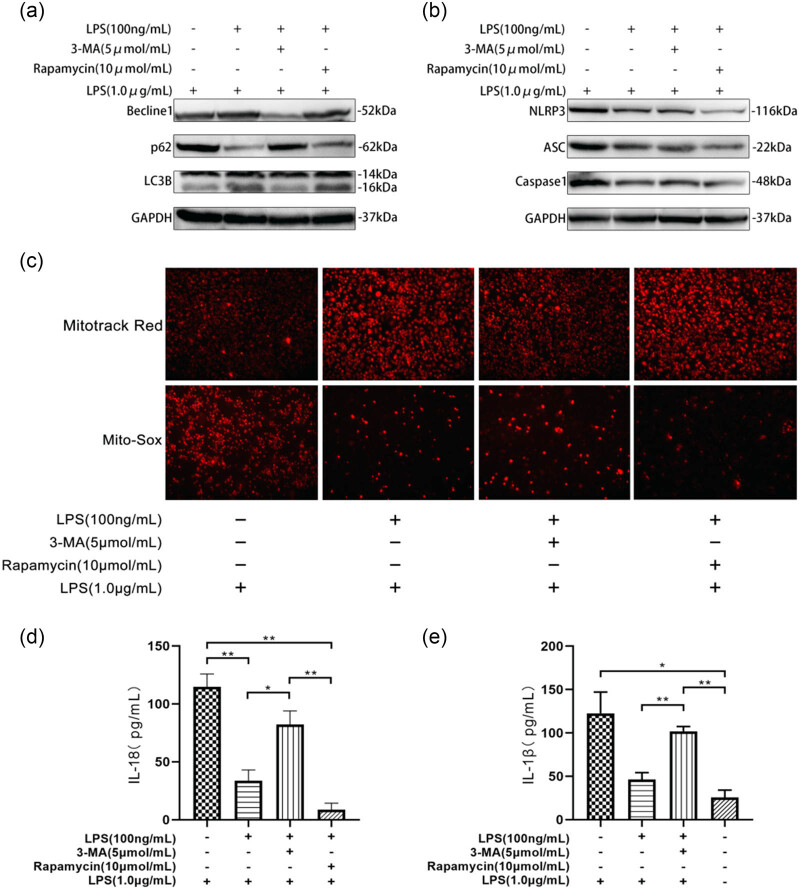
Inhibition of autophagy can reverse the protective effect of ET on LPS-stimulated RAW264.7 cells. RAW264.7 cells were pretreated with LPS (100 ng/mL) for 24 h, washed three times with PBS, and then treated with rapamycin (10 µmol/mL) for 48 h or 3-MA (5 µmol/mL) for 4 h, washed with PBS for once again, stimulated with LPS (1 µg/mL) for 24 h. (a) Western blot analysis of Beclin1, P62, and LC3B autophagy-related protein expression levels in RAW264.7 cells. (b) Western blot analysis of NLRP3, ASC, and Caspase-1 protein expression levels in RAW264.7 cells. (c) MitoTracker Red probe was used to measure mitochondrial membrane potential and Mito-Sox was used to measure mtROS in RAW264.7 cells. (d) The level of IL-18 in the culture supernatant of RAW264.7 cells by ELISA. (e) The levels of IL-1β in the culture supernatant of RAW264.7 cells by ELISA.*P < 0.05, **P < 0.01.

To explore the relationship between ET, activation of the NLRP3 inflammasome, mitochondrial damage in macrophages, and autophagic flux, we divided RAW264.7 macrophages into control, LPS (100 ng/mL) pretreatment, 3-MA, and rapamycin groups, and treated the cells with LPS (1 μg/mL) for 24 h. The results showed that compared to the control group, the LPS pretreatment group exhibited significantly reduced ROS levels and mitochondrial transmembrane potential damage (Figure 4c), as well as increased LC3BII/LC3B-I and Beclin-1 levels, with a decrease in P62 (Figure 4a), indicating enhanced autophagic activity. Additionally, the expression of NLRP3, ASC, and caspase-1 proteins, as well as the levels of IL-1β and IL-18 in the cell culture supernatant, were significantly reduced (Figure 4b, d and e), suggesting that ET inhibits the activation of the NLRP3 inflammasome. These findings indicate that ET alleviates mitochondrial damage in macrophages by restoring autophagic flux and suppressing NLRP3 inflammasome activation, further corroborating previous research.

However, when autophagy was inhibited by 3-MA, the beneficial effects of ET were suppressed. This resulted in an increase in ROS levels and mitochondrial transmembrane potential damage, along with lower levels of LC3BII/LC3B-I and Beclin-1, and P62 accumulation compared to the LPS pretreatment group. Furthermore, the expression of NLRP3, ASC, and caspase-1 proteins, as well as the levels of IL-1β and IL-18, were significantly elevated, indicating that autophagy inhibition restored NLRP3 inflammasome activation.

Moreover, in the rapamycin group, which enhanced autophagy, the protective effects of ET were further potentiated. Specifically, ROS levels and mitochondrial transmembrane potential damage were significantly reduced, LC3BII/LC3B-I and Beclin-1 increased, P62 decreased, and the expression of NLRP3, ASC, and caspase-1 proteins, along with the levels of IL-1β and IL-18, were significantly lower. These results further support the conclusion that autophagy restoration helps to suppress NLRP3 inflammasome activation.

In summary, ET restores autophagic flux in RAW264.7 cells, reduces mitochondrial damage and ROS generation, inhibits NLRP3 inflammasome activation, and protects macrophages from LPS-induced damage. The autophagy inhibitor 3-MA suppressed this protective effect, whereas the autophagy enhancer rapamycin further enhanced it. These findings suggest that ET inhibits macrophages in septic mice through the restoration of autophagic flux and suppresses NLRP3 inflammasome activation via this mechanism.

### 3.4 Key role of TRIM26 in ET

Based on the experiments outlined above, we demonstrate that ET plays a crucial role through autophagy. Which genes are involved in regulating this process is a key focus of our further investigation. Through a thorough review of the literature, we identified that TRIM26 plays an important role in immune responses and the regulation of autophagy. TRIM26 is an E3 ubiquitin ligase that has been shown to be critical in various cellular processes, particularly in regulating immune responses and autophagy. Studies suggest that TRIM26, through its ubiquitination activity, regulates the stability of autophagy-related proteins (such as Beclin-1 and LC3), thereby influencing the initiation and completion of autophagic flux [36]. Additionally, TRIM26 is closely associated with the activation of the NLRP3 inflammasome. By affecting the degradation of autophagy-related proteins, it indirectly regulates the activation of the NLRP3 inflammasome, thus modulating the intensity of immune responses [37]. These studies suggest that TRIM26 may play a pivotal regulatory role in ET.

**Figure 5:**
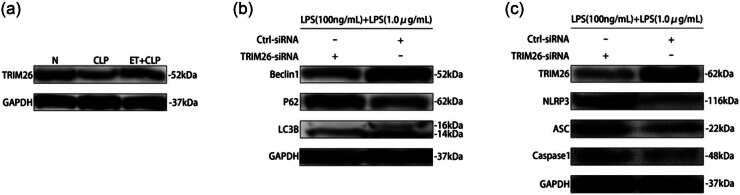
Key role of TRIM26 in ET. (a) TRIM26 protein expression levels in primary macrophages by western blot. (b) Autophagy-related protein Beclin1, P62, and LC3B expression level by western blot in tolerant RAW264.7 cells transfected with control-siRNA or TRIM26-siRNA. (c) TRIM26, NLRP3, ASC, and Caspase-1 protein expression level by western blot in tolerant RAW264.7 cells transfected with control-siRNA or TRIM26-siRNA.

Our experimental results indicate that in primary macrophages from septic mice induced by LPS (1 μg/mL), the protein expression level of TRIM26 was downregulated compared to the control group. However, in the endotoxin-tolerant group established by pre-treatment with LPS (100 ng/mL), the downregulation of TRIM26 was attenuated (Figure 5a). Therefore, we aim to explore whether ET regulates autophagy through the key gene TRIM26.

To investigate this, we first established ET in RAW264.7 cells using LPS (100 ng/mL) and transfected the cells with either control-siRNA or TRIM26-siRNA. As shown in Figure 5b and c, the protein expression of TRIM26 was reduced in TRIM26-siRNA-treated cells. Following treatment with LPS (1 μg/mL) in these transfected cells, our results revealed that in endotoxin-tolerant macrophages, lowering TRIM26 expression resulted in the following changes: accumulation of P62, a hallmark of autophagy blockage, indicating impaired autophagic function. Furthermore, there was an increase in the activation of NLRP3 inflammasome-related proteins. These results suggest that ET regulates the activation of the NLRP3 inflammasome through autophagy, with TRIM26 playing a critical role in the autophagic regulation of ET.

## 4 Discussion

Sepsis affects over 31 million people worldwide annually and is one of the leading causes of death among critically ill patients. However, there are currently no approved molecular therapies for sepsis [38]. Inflammatory imbalance is a key factor in the pathogenesis of sepsis and occurs throughout its course. The host’s initial acute response to pathogenic microorganisms typically leads to macrophage phagocytosis of pathogens and the production of a series of pro-inflammatory cytokines, which can trigger a cytokine storm, activating the innate immune system [3]. Macrophage ET is a state that occurs following low-dose LPS stimulation; after exposure to endotoxins, the levels of inflammatory mediators produced upon re-stimulation with LPS are reduced. ET is an important regulatory mechanism that controls excessive inflammatory responses [39]. However, how endotoxin-tolerant macrophages downregulate inflammatory cytokines remain unclear. Therefore, the primary objective of this study was to elucidate how ET regulates autophagic flux through TRIM26, thereby inhibiting the activation of NLRP3 inflammasomes in macrophages from septic mice.

We found that ET reduces the levels of pro-inflammatory cytokines in septic mice and alleviates inflammatory damage in various organs. The protective effects of ET were observed not only in septic mice but also in primary mouse peritoneal macrophages that had been made tolerant through low-dose LPS stimulation, where the levels of TNF-α and IL-1β were significantly reduced. This finding is consistent with previous studies [40]. The expression of IL-1β is closely associated with the activation of the NLRP3 inflammasome [41]. We assessed the expression of the NLRP3 inflammasome and found that, under high-dose LPS stimulation, ET reduced the activation of the NLRP3 inflammasome in macrophages. It is currently believed that the activation of the NLRP3 inflammasome may be related to potassium ion efflux, lysosomal rupture, and excessive release of ROS [42]. Furthermore, LPS promoted ROS production in macrophages. ROS production within cells primarily originates from mitochondria and the NADPH oxidase (NOX) system on the cell membrane, and mtROS generation is closely associated with the development of sepsis [43].

Furthermore, we found that the activation of the NLRP3 inflammasome activates autophagy. In this study, we found that the expression of inflammatory cytokines, ROS production, autophagy, and apoptosis were increased in LPS-stimulated macrophages. Notably, Beclin1 and LC3BII/I levels were also increased. However, LPS stimulation also induced P62 accumulation (Figure 3). ET reduces ROS production, mitochondrial damage, apoptosis, and P62 accumulation. P62 is the most important ubiquitin-binding protein in autophagosomes. During autophagy, P62 recognizes and binds to ubiquitinated proteins, forms a complex with ASC, and degrades damaged organelles and misfolded proteins using the autophagy-lysosome system. The accumulation of P62 often occurs when autophagy is inhibited. Therefore, P62 is considered a marker which reflects autophagic activity [44]. These results indicate that macrophages stimulated by LPS exhibit autophagy activation but impaired autophagic flux, whereas ET can restore autophagic flux.

In addition, we found that the activation of the NLRP3 inflammasome promotes autophagy. In this study, LPS-stimulated macrophages showed increased levels of pro-inflammatory cytokines, ROS production, autophagy, and apoptosis. Notably, the levels of Beclin1 and LC3BII/I were also increased. However, LPS stimulation also caused the accumulation of P62. ET reduced ROS generation, mitochondrial damage, apoptosis, and P62 accumulation. P62 is the most important ubiquitin-binding protein in autophagosomes. During autophagy, P62 recognizes and binds to ubiquitinated proteins, forming complexes with ASC and facilitating the degradation of damaged organelles and misfolded proteins via the autophagy-lysosome system. The accumulation of P62 usually occurs when autophagy is blocked, making it a marker for autophagic activity [45]. These results suggest that LPS-stimulated macrophages exhibit activated autophagy but with blocked autophagic flux, whereas ET restores autophagic flux.

Recent studies have shown that autophagy regulates inflammasome formation [46]. Autophagy inhibits the assembly of the NLRP3 inflammasome, thus reducing LPS-induced inflammatory responses in mouse macrophages [47].

To further investigate the relationship between ET and NLRP3 inflammasome activation, we analyzed the expression of NLRP3 inflammasomes following autophagy enhancement or suppression. We observed that ET enhanced autophagy and reduced inflammation. The expression of NLRP3 inflammasomes further suppressed or enhanced autophagy, and the activation of NLRP3 inflammasomes correspondingly increased or decreased. This suggests that ET may suppress NLRP3 inflammasome activation by enhancing autophagy. During the pathogenesis of sepsis, macrophages are exposed to various environmental stresses, including oxidative stress, which may induce the formation of NLRP3 inflammasomes. ET increases autophagy, thereby reducing mtROS generation and damage to macrophage mitochondrial membrane potential. This study also observed significant increase in intracellular ROS under pathological conditions. These results suggest that mtROS may have an interplay with ET. However, the mechanisms by which ET reverses mtROS production remain unclear.

In summary, our findings indicate that ET enhances autophagic flux, suppresses NLRP3 inflammasome activation, and mitigates excessive inflammatory responses in sepsis. Targeting key genes in this process is crucial for the development of therapeutic interventions. Literature suggests that TRIM26 plays a pivotal role in regulating autophagy [48,49]. As an E3 ubiquitin ligase, TRIM26 regulates its antifibrotic effects by promoting the ubiquitination and proteasomal degradation of SLC7A11, enhancing ferroptosis in activated hepatic stellate cells, and exerting antifibrotic effects in the liver [50]. TRIM26 contains an ubiquitin-binding region, enabling P62 to bind polyubiquitin chains, recognize ubiquitinated substrates, and transport them to autophagosomes [51]. Considering the critical role of P62 in ubiquitination-mediated autophagy, we explored the relationship between P62 and the ubiquitin ligase TRIM26. We observed that silencing TRIM26 led to P62 accumulation, which impaired autophagic flux, downregulated ubiquitination, and facilitated the degradation of NLRP3 inflammasomes in macrophages. Previous studies have shown that TRIM26, as a major E3 ubiquitin ligase, targets NTH1 for ubiquitination and proteasomal degradation [52]. Therefore, TRIM26 protects mice from LPS-induced sepsis via ubiquitination. This study provides new insights into how ET regulates autophagy via TRIM26-mediated ubiquitination and reveals the novel function of TRIM26 in regulating the innate immune response.

## 5 Limitations

Despite the novel findings in this study, there are two major limitations. First, although we have demonstrated that ET reduces the activation of NLRP3 inflammasomes, mtROS generation, and membrane potential damage, restores autophagic flux, and suppresses apoptosis, the relationship between these effects and mitochondrial autophagy remains unclear. Second, the protective effects of ET on macrophages may also be influenced by other signaling pathways in addition to the restoration of autophagic flux and NLRP3 inflammasome activation. Therefore, future studies should include signaling pathways such as the PTEN pathway to further explore the protective effects of ET in septic mice.

## 6 Conclusion

ET restores autophagic flux in macrophages, suppresses NLRP3 inflammasome activation, and alleviates inflammatory damage in septic mice, possibly through the regulatory effects of TRIM26.
